# Supplementary material for: Content-rich biological network constructed by mining PubMed abstracts
Source: BMC Bioinformatics. 2004 Oct 8;5:147. doi: 10.1186/1471-2105-5-147 (PMC528731; doi:10.1186/1471-2105-5-147)
Supplement: Additional File 2 — The original results of the above study (non-essential files are deleted to keep the file size under the limit set by BMC bioinformatics). [file 1471-2105-5-147-S2.bz2 › chilibotAdditionalFile2/dip30/699ID7726836E18027/html/FTSZ_SULA.html]

 


 **FTSZ** and **SULA** 
  
Found 31 abstracts in PubMed, retrieved 30.  
 

 What does Google say? 
 PDF only 
| .edu only 

---

**Interactive relationship** (e.g. stimulation, inhibition, etc)

**Inhibitory relationship**- Bacterial SOS checkpoint protein  **SulA**  inhibits polymerization of purified  **FtsZ**  cell division protein.  Ref: 9683493 J Bacteriol, 1998
- Role of the SulB  **FtsZ**  protein in division inhibition during the SOS response in Escherichia coli  **FtsZ**  stabilizes the inhibitor  **SulA**  in maxicells.  Ref: 2994059 Proc Natl Acad Sci U S A, 1985
- **SulA**  prevented the formation of high molecular weight  **FtsZ**  polymers by interfering with  **FtsZ**  dimerization and subsequent oligomerization.  Ref: 10931335 Mol Microbiol, 2000
- We propose that the  **SulA**  inhibitor interacts directly with  **FtsZ**  in vivo to block the essential division function of this protein.  Ref: 2994059 Proc Natl Acad Sci U S A, 1985
- This interaction was reduced by mutations in  **sulA**  and by most mutations in  **ftsZ**  that make cell refractory to  **sulA** .  Ref: 8752322 J Bacteriol, 1996
- Cell division inhibitors  **SulA**  and MinCD prevent formation of the  **FtsZ**  ring.  Ref: 8432706 J Bacteriol, 1993
- A cell division inhibitor  [ **SULA** ]   **SulA**  of Escherichia coli directly interacts with  **FtsZ**  through GTP hydrolysis.  Ref: 7726836 Biochem Biophys Res Commun, 1995
- Mutations in the essential cell division gene  **ftsZ**  confer resistance to  **SulA** , a cell division inhibitor  [ **SULA** ]  that is induced as part of the SOS response.  Ref: 8282688 J Bacteriol, 1994
- Reversal of  **SulA**  inhibition by blocking protein synthesis in  **SulA**  induced filaments led to a resumption of  **FtsZ**  ring formation and division.  Ref: 8432706 J Bacteriol, 1993
- The classic SOS gene product that inhibits cell division is SfiA or  **SulA** , which binds to  **FtsZ**  and prevents septum formation until the DNA damage has been repaired.  Ref: 9068638 J Bacteriol, 1997
- Instead,  **SulA**  binds the T7 loop surface of  **FtsZ** , opposite the nucleotide binding site, blocking polymer formation.  Ref: 12808143 Proc Natl Acad Sci U S A< MedlineTA>Proc Natl Acad Sci U S A, 1997
- Analysis of  **ftsZ**  mutations that confer resistance to the cell division inhibitor  [ **SULA** ]   **SulA**  SfiA.  Ref: 2145263 J Bacteriol, 1990
- This possibility was supported by the finding that alleles of  **ftsZ**  isolated as resistant to the cell division inhibitor  [ **SULA** ]   **SulA**  were also resistant to MinCD.  Ref: 2211499 J Bacteriol, 1990
- A lacZ  **ftsZ**  gene fusion is an analog of the cell division inhibitor  [ **SULA** ]   **sulA** .  Ref: 6230346 J Bacteriol, 1984
- **SulA**  protein inhibits the polymerization and the GTPase activity of  **FtsZ** , while point mutant  **SulA**  proteins show little effect on either of these  **FtsZ**  activities.  Ref: 9683493 J Bacteriol, 1998
- Furthermore, strains carrying various alleles of  **ftsZ** , selected on the basis of resistance to the inhibitor  **SulA** , displayed variable resistance to MinCD.  Ref: 11673433 J Bacteriol, 2001
- Whereas the  **ftsZ**  mutations were identical to a mutation known to produce a protein refractory to  **SulA**  inhibition, the  **sulA**  mutations affected the promoter operator region, reducing synthesis of  **SulA** .  Ref: 10368140 J Bacteriol, 1999
- It is proposed that when overproduced, the HslV HslU protease complex can degrade  **SulA**  which is an endogenous inhibitor of the essential cell division protein  **FtsZ**   [ **FTSZ** ] .  Ref: 9315728 FEBS Lett, 1997
- These results indicate that MinCD blocks the assembly of the septation machinery at a later step than  **SulA** , at the stage at which FtsA is added to the  **FtsZ**  ring.  Ref: 10931335 Mol Microbiol, 2000
- **SulA**  is induced in Escherichia coli by the SOS response and inhibits cell division through interaction with  **FtsZ** .  Ref: 9180687 Mol Gen Genet, 1997
- Several dominant mutations that make lon mutant cells refractory to the cell division inhibitor  [ **SULA** ]   **SulA** , sulB9, sulB25, and sfiB114, have been mapped to the  **ftsZ**  gene.  Ref: 2145263 J Bacteriol, 1990
**Stimulatory relationship**- These results suggest that  **SulA**  inhibits cell division by inhibiting  **FtsZ** .  Ref: 3011743 J Bacteriol, 1986
- These results indicate that  **SulA**  blocks Z ring formation by blocking  **FtsZ**  polymerization.  Ref: 9501185 Proc Natl Acad Sci U S A, 1998
- The revertants were found to carry a suppressor affecting either  **ftsZ**  encoding a key cell division protein or  **sulA**  encoding the  **SulA**  inhibitor, which binds and inhibits  **FtsZ** .  Ref: 10368140 J Bacteriol, 1999
- Here we show that a MalE  **SulA**  fusion that retains this inhibitory function in vivo inhibits the GTPase activity and polymerization of  **FtsZ**  in vitro.  Ref: 9501185 Proc Natl Acad Sci U S A, 1998
- **FtsZ**  Rsa mutant proteins have an increased ability for multimerization, making them resistant to  **SulA**  and ZZ.  Ref: 2145263 J Bacteriol, 1990
- Induction of the cell division inhibitor  [ **SULA** ]   **SulA** , a component of the SOS response, or the inhibitor MinCD, a component of the min system, blocked formation of the  **FtsZ**  ring and led to filamentation.  Ref: 8432706 J Bacteriol, 1993
**Neutral relationship**- An interaction was observed between  **FtsZ**  and  **SulA** , a component of the SOS response, and the interacting regions were mapped to their conserved domains.  Ref: 8752322 J Bacteriol, 1996
- It is likely that mutations in  **ftsZ**  that confer  **sulA**  resistance alter the conformation of the protein such that it assumes the active form.  Ref: 8282688 J Bacteriol, 1994
- Mutations in  **ftsZ**  that confer resistance to  **SulA**  affect the interaction of  **FtsZ**  with GTP.  Ref: 8282688 J Bacteriol, 1994
- An increased gene dosage of  **ftsZ**  or the presence of the sulB allele of  **ftsZ**  is known to overcome  **sulA**  mediated inhibition of division during the SOS response.  Ref: 6230346 J Bacteriol, 1984
- The  **SulA**  protein interacts with the tubulin like  **FtsZ**  division protein.  Ref: 9683493 J Bacteriol, 1998
- Using purified  **SulA**  protein that was fused to the maltose binding protein, we demonstrate in vitro that  **SulA**  interacts with  **FtsZ**  to form a stable complex.  Ref: 7726836 Biochem Biophys Res Commun, 1995
- They may, instead, decrease the  **SulA**   **FtsZ**  interaction.  Ref: 2254289 J Bacteriol, 1990
- In addition to being resistant to  **SulA** , all  **ftsZ**  Rsa mutations also conferred resistance to a LacZ  **FtsZ**  hybrid protein ZZ.  Ref: 2145263 J Bacteriol, 1990
- Addition of the  **SulA**  protein to the assay leads to rapid disassembly of existing  **FtsZ**  polymers, demonstrating its natural regulatory effect on  **FtsZ**  under the assay conditions.  Ref: 12202250 Anal Biochem, 2002
- DNA sequence analysis of these mutations and the sfiB103 mutation confirmed that all of these mutations mapped within the  **ftsZ**  gene and revealed that the two sulB mutations were identical and by selection for resistance to higher levels of  **SulA** , contained a second mutation within the  **ftsZ**  gene.  Ref: 2145263 J Bacteriol, 1990
- **SulA**  halts cell division in Escherichia coli by binding to the major component of the division machinery  **FtsZ** .  Ref: 12808143 Proc Natl Acad Sci U S A< MedlineTA>Proc Natl Acad Sci U S A, 1990

**Non-interactive relationship** (e.g. studied together, co-existance, homology, etc.)

- In the presence of  **FtsZ** , the half life of  **SulA**  synthesized in maxicells is approximately 12 min.  Ref: 2994059 Proc Natl Acad Sci U S A, 1985
- We therefore propose that these mutations be redesignated  **ftsZ**  Rsa for resistance to  **SulA** .  Ref: 2145263 J Bacteriol, 1990
- We investigated the effects of purified  **SulA**  upon  **FtsZ** .  Ref: 9683493 J Bacteriol, 1998
- **SulA**  inhibits cell division neither by binding the nucleotide binding site nor by inducing conformational changes in  **FtsZ** .  Ref: 12808143 Proc Natl Acad Sci U S A< MedlineTA>Proc Natl Acad Sci U S A, 1998
- Crystal structure of the SOS cell division inhibitor  [ **SULA** ]   **SulA**  and in complex with  **FtsZ** .  Ref: 12808143 Proc Natl Acad Sci U S A< MedlineTA>Proc Natl Acad Sci U S A, 1998
- Both  **FtsZ**  and  **SulA**  are isolated with the inner membrane of E. coli maxicells in the presence of MgCl2.  Ref: 2994059 Proc Natl Acad Sci U S A, 1985
- The cell division protein  **FtsZ**   [ **FTSZ** ] , target of the SOS associated division inhibitor SfiA or  **SulA** , seems to be involved in the postshift division delay.  Ref: 3040674 J Bacteriol, 1987
- In contrast, in the absence of  **FtsZ**  or in the presence of a mutant form of  **FtsZ**  SulB114 that prevents division inhibition in vivo,  **SulA**  is extremely unstable with a half life of only 3 min.  Ref: 2994059 Proc Natl Acad Sci U S A, 1985
- Analysis of mutations within the  **ftsZ** , gene, selected for resistance to the cell division inhibitor  [ **SULA** ]   **SulA** , revealed that they also conferred resistance to MinCD.  Ref: 1925024 Res Microbiol< MedlineTA>Res Microbiol, 1985
- We have solved the crystal structure of  **SulA**  alone and in complex with  **FtsZ**  from Pseudomonas aeruginosa.  Ref: 12808143 Proc Natl Acad Sci U S A< MedlineTA>Proc Natl Acad Sci U S A, 1985
- direct, reversible inhibition of  **FtsZ**  polymerization by  **SulA**  may account for division inhibition.  Ref: 9683493 J Bacteriol, 1998
- MinC does not act like  **SulA** , which affects the GTPase activity of  **FtsZ** , and the model can explain how MinC would select for the  **FtsZ**  polymer rather than the monomer.  Ref: 11350934 EMBO J, 2001
- Inhibition of  **FtsZ**  polymerization by  **SulA** , an inhibitor of septation in Escherichia coli.  Ref: 9501185 Proc Natl Acad Sci U S A, 1998
- In this paper, size exclusion chromatography was used to study the effect of the  **SulA**  and MinCD division inhibitors on the oligomerization state of endogenous  **FtsZ**  in cytoplasmic extracts, and immunofluorescence microscopy was used to determine the effect of  **SulA**  and MinCD on the formation of  **FtsZ** , FtsA and ZipA rings at potential division sites.  Ref: 10931335 Mol Microbiol, 2000
- **SulA**  overproducing cells had a lysis positive phenotype, the ring structure but not the GTPase function of  **FtsZ**  was impaired.  Ref: 9732440 Arch Microbiol, 1998
- The block in initiation of division in the ftsW201 allele is shown to be independent of  **FtsZ**  or the  **FtsZ**  inhibitor,  **SulA** .  Ref: 7961485 J Bacteriol, 1994
- Of these genes,  **ftsZ**  is of great interest as it acts earliest in the division pathway, is essential, its level dictates the frequency of division, and it is thought to be the target of two cell division inhibitors,  **SulA** , produced in response to DNA damage, and MinCD, which prevents division at old sites.  Ref: 1944597 Nature, 1991
